# Estimating the impact of multiple immunization products on medically-attended respiratory syncytial virus (RSV) infections in infants

**DOI:** 10.1016/j.vaccine.2019.10.023

**Published:** 2019-11-16

**Authors:** Gabriel Rainisch, Bishwa Adhikari, Martin I. Meltzer, Gayle Langley

**Affiliations:** a US Centers for Disease Control and Prevention (CDC), Atlanta, GA, USA; b National Center for Emerging and Zoonotic Infectious Diseases (NCEZID), USA; c National Center for Immunization and Respiratory Diseases (NCIRD), USA

**Keywords:** RSV, Maternal vaccination, Passive immunization, Model, Infants, United States

## Abstract

**Background:**

Palivizumab, a monoclonal antibody and the only licensed immunization product for preventing respiratory syncytial virus (RSV) infection, is recommended for children with certain high-risk conditions. Other antibody products and maternal vaccines targeting young infants are in clinical development. Few studies have compared products closest to potential licensure and have primarily focused on the effects on hospitalizations only. Estimates of the impact of these products on medically-attended (MA) infections in a variety of healthcare settings are needed to assist with developing RSV immunization recommendations.

**Methods:**

We developed a tool for practicing public health officials to estimate the impact of immunization strategies on RSV-associated MA lower respiratory tract infections (LRTIs) in various healthcare settings among infants <12 months. Users input RSV burden and seasonality and examine the influence of altering product efficacy and uptake assumptions. We used the tool to evaluate candidate products’ impacts among a US birth cohort.

**Results:**

We estimated without immunization, 407,360 (range: 339,650–475,980) LRTIs are attended annually in outpatient clinics, 147,240 (126,070–168,510) in emergency departments (EDs), and 33,180 (24,760–42,900) in hospitals. A passive antibody candidate targeting all infants prevented the most LRTIs: 196,470 (48% of visits without immunization) outpatient clinic visits (range: 163,810–229,650), 75,250 (51%) EDs visits (64,430–86,090), and 18,140 (55%) hospitalizations (13,770–23,160). A strategy combining maternal vaccine candidate and palivizumab prevented 58,210 (14% of visits without immunization) LRTIs in outpatient clinics (range: 48,520–67,970), 19,580 (13%) in EDs (16,760–22,400), and 8,190 (25%) hospitalizations (6,390–10,150).

**Conclusions:**

Results underscore the potential for anticipated products to reduce serious RSV illness. Our tool (provided to readers) can be used by different jurisdictions and accept updated data. Results can aid economic evaluations and public health decision-making regarding RSV immunization products.

## 1. Introduction

Globally, respiratory syncytial virus (RSV) is a leading cause of severe respiratory tract infections among young children. In 2015, there were an estimated 33.1 million acute lower respiratory tract infections, 3.2 million hospital admissions and 59,600 in-hospital deaths attributed to RSV infections (RSVi) among children < 5 years of age worldwide. About 45% of RSV-associated hospitalizations and deaths occurred among children <6 months of age [1]. Each year in the United States, ~1.5 million outpatient visits, ~500,000 emergency department (ED) visits, ~58,000 hospitalizations and ~150 deaths are associated with RSVi among children under 5 years of age [2,3]. Rates of medically-attended RSVi (MA-RSVi) in the United States are highest amongst infants < 6-months of age [4,5]. In the US and other temperate climates, RSV season generally lasts six months between fall and spring with a peak during the winter [6]. In countries with tropical or subtropical climates, the season may be longer and less predictable [7].

Palivizumab, currently the only licensed product to prevent RSVi, is recommended for use in children with certain “high risk” conditions [8]. It is given in monthly intramuscular injections during RSV season. There are over 40 vaccine and antibody products in development for prevention of RSVi [9]. Two products in late stages of clinical development target young infants: (1) a monoclonal antibody designed to provide direct protection (completed phase 2b clinical trial) [10]; and (2) a maternal vaccine designed to provide indirect protection through passive placental transfer of antibodies (completed phase 3 clinical trial) [11]. Both of these products aim to protect against medically-attended lower respiratory tract infections (MA-LRTI) due to RSV. Additional maternal vaccines and antibody products are in the clinical development pipeline [9].

Previous studies have evaluated the potential impacts of immunization on MA-RSVi in a variety of countries [12–19]. These analyses have focused on the hospital setting and impacts from single, theoretical vaccine products. Only one (Cromer et al.) simultaneously compared multiple products in the later stages of clinical development and across several healthcare settings [13]. Cromer et al. estimated the direct effects of various pediatric and maternal immunization candidate products and strategies using a cohort model in England. While Cromer et al.’s model more closely matches trial endpoints for products potentially close to licensure, its assumptions may not be generalizable to populations that have different rates of disease and seasonality. It also assumed the entire population eligible for an immunization product received it (i.e. 100% uptake), which likely overestimates the public response. The evolving state of product development highlights the need for flexible and accessible modeling tools, which can be readily updated to reflect advancements in our knowledge of product characteristics, and which can be applied to jurisdictions with varied RSV epidemiology.

We therefore developed a modeling tool, called the RSV Immunization Impact Model (RSV I^2^M), for use by practicing public health officials and policy-makers in their jurisdictions, to estimate the direct effects of immunization candidates targeting young infants, on MA-RSV-associated LRTIs. RSV I^2^M evaluates the potential impact of these products on outpatient clinic visits, ED visits, and hospitalizations based on user-adjustable RSVi rates and seasonality, in conjunction with assumptions about product uptake and efficacy. Model outputs (visits with and without immunization for LRTI due to RSV) can assist policy-makers in the United States and other countries with developing economic analyses and recommendations for RSV immunization. We also apply the model to a US birth cohort to estimate the potential impact of these products on MA-LRTI due to RSV in the United States.

## 2. Methods

### 2.1. Tool overview

RSV I^2^M is a spreadsheet-based tool (Supplementary Material [S1]) that uses a Decision Tree model to estimate the potential impact of three immunization strategies on MA-RSV-associated LRTIs among an annual birth cohort through 12 months of age. The birth cohort is divided into “high-risk” and “low-risk” (all other) infants. High-risk infants include those with hemodynamically significant CHD, chronic lung disease of prematurity (CLDP), and infants born prematurely at <29 weeks gestational age based on recommendations for who should receive palivizumab prophylaxis [8]. The first strategy (Strategy I) generally follows current US-based recommendations that high-risk infants receive monthly injections of palivizumab during the RSV season (typically October to March) during their first year of life. [8] In the model, palivizumab is given starting at birth for those born during the season, and starting at the beginning of the next RSV season when births occur out-of-season (OoS) (Table 1). The second strategy (Strategy II) provides a new antibody product, hereafter referred to as the “Antibody Candidate” strategy, injected as a single dose with the same timing of palivizumab initiation, but targeting all infants rather than just those at high risk. The third strategy (Strategy III), the “Maternal Vaccine Candidate + Palivizumab” strategy, combines providing vaccine to mothers in their third trimester throughout the year (not just during the season) and palivizumab to high-risk births based on the palivizumab schedule described above.

Estimates of MA-RSVi visits without any immunization are based upon user inputs regarding the size of their birth cohort, prevalence and risk of RSV hospitalizations among those with high-risk conditions, rates of RSV (combined for high- and low-risk infants) by month of age in the outpatient clinic, ED, and hospital settings, the proportion of MA-RSVi visits resulting in a LRTI diagnosis, and RSV seasonality (Table 2). To estimate the effects of immunization, users input immunization uptake, efficacy, and duration of protection for each product (Table 2). Uptake was defined as the proportion of the population expected to receive the products. Efficacy is defined as the percent protected assuming recipients receive the full immunization dose at the correct time. For the maternal vaccine, efficacy is reduced by assumptions about the proportion of antibodies that successfully transfer to the infant (based on the timing of the mother’s vaccination and the infant’s gestational age a birth; Table 2, Supplementary Material [S2]). Users can readily update a number of input values as new data become available or to reflect a jurisdiction’s immunization policy considerations. To illustrate the tool, we used it to estimate the effects of the aforementioned immunization strategies on a US birth cohort.

### 2.2. Calculations

#### 2.2.1. Visits without immunization

To calculate the number of MA-RSVi resulting in LRTI for each of the three healthcare settings, we multiplied the “all-risk” (high-and low-risk) MA-RSVi rate by the proportion of visits in each setting with an LRTI diagnosis and the size of monthly birth cohorts (assuming births occur evenly across the year) (S2). These results were then distributed to calendar months based on RSV seasonality by multiplying them by the percent of annual visits occurring in each month. For countries that currently use palivizumab, like the United States, we added to the monthly visit counts MA-LRTIs that would have occurred in the absence of palivizumab. For the hospital setting, these additional visits were determined by multiplying the rate of hospitalizations among high-risk infants by the size of the high-risk cohort, palivizumab uptake, and palivizumab efficacy. The hospitalization rates used in this calculation are a weighted average across the different high-risk groups (S2). For the outpatient clinic and ED settings, we assumed the ratio of rates between high- and low-risk infants is the same as the ratio of hospitalization rates for high and low risk infants, and that palivizumab would have the same efficacy for preventing cases in these settings (S2).

#### 2.2.2. Visits prevented with immunization

To obtain the annual number of visits prevented with immunization for a given strategy and setting, we summed the visits prevented across all months that the immunization remained protective, based on its duration of protection. We calculated the monthly visits prevented with each immunization strategy differently. For Strategy I, LRTI visits prevented by palivizumab equaled the calculated number of MA-RSV-associated LRTIs without immunization among high-risk infants, multiplied by palivizumab uptake and efficacy. For Strategies II and III, visits prevented by the immunization candidates equaled the number of MA-RSV-associated LRTIs without immunization among both high- and low-risk infants, multiplied by the candidate uptake in each risk group and efficacy. The efficacies for both candidates assumes recipients receive the full immunization dose. To account for incomplete transfer of antibodies from mother to child for a portion of births, we multiplied the maternal vaccine efficacy in Strategy III by a reduction factor. This factor considers the delay in the mother’s production of antibodies after vaccination (dependent on the timing of vaccination relative to birth) and the fact that the amount of antibody transfer is dependent on gestational age at birth (S2). In Strategy III, high-risk infants are also eligible to receive palivizumab; therefore, we added the number of visits prevented by palivizumab when calculating the total annual prevented visits for this strategy. Finally, we calculated the visits that would occur despite having each immunization strategy in place: this equaled visits without immunization minus visits prevented.

#### 2.2.3. Deaths with and without immunization

Since data are sparse on the number of RSV-associated deaths that occur outside the hospital setting, we estimated deaths with and without immunization based on deaths among hospitalized infants. We calculated deaths without immunization by multiplying user-provided hospitalized case fatality ratios (hCFR) for infants 0–5 months of age and for those between 6 and 11 months of age and the total annual estimate for hospitalizations due to RSV-associated LRTIs without immunization for these age groups. Deaths prevented by immunization were calculated similarly to medically-attended visits prevented, whereby deaths that occur without immunization were multiplied by the uptake and efficacy for each product. Finally, deaths that would occur despite having each immunization strategy in place equaled deaths without immunization minus deaths prevented through immunization.

### 2.3. Model inputs and sensitivity analysis

To illustrate the model, we estimated the impact of implementing the three immunization strategies in the United States. Table 2 includes all model inputs, values used and sources (with additional detail in S2).

We conducted two sensitivity analyses of immunization candidates’ impacts. In the first, we evaluated the influence of high and low estimates for individual parameters, while all other parameters were held constant. For this analysis we used the 95% CI bounds for MA-RSVi rates, five percentage point reductions and improvements in the baseline uptake for the antibody candidate (66–76%) and maternal vaccine candidate (51–61%), the 95% CI bounds for efficacy reported in clinical trial results for an antibody candidate (73–85%, which we assumed for the maternal vaccine candidate as well), and one month reductions and improvements in durations of the antibody candidate (120–180 days), and maternal vaccine candidate (60–120 days) (Table 2).

In our second sensitivity analysis, we examined the impact of uptake of the immunization candidates on LRTI visits by accounting simultaneously for uncertainty in RSV rates, uptake, efficacy, and duration. We present the results for this analysis as the lowest and highest possible prevented visits associated with a percentage point decrease or increase in uptake, respectively. We generated the lowest estimate by combining the 2.5 percentile values for MA-RSVi rates, lowest efficacy and uptake, and shortest duration, for each product as inputs (Table 2). High estimates were achieved by combining the 97.5 percentile values for MA-RSVi rates, highest efficacy and uptake, and longest duration, for each product.

## 3. Results

### 3.1. Visits without immunization

We estimate, in the absence of palivizumab use, RSV-associated LRTIs in the US among infants up to 12 months of age, would result in 407,360 annual outpatient clinic visits (range, based on RSV rates uncertainty: 339,650–475,980); 147,240 annual ED visits (range: 126,070–168,510), and annual 33,180 hospitalizations (range: 24,760–42,900).

### 3.2. Visits prevented with immunization

**Fig. 1.**
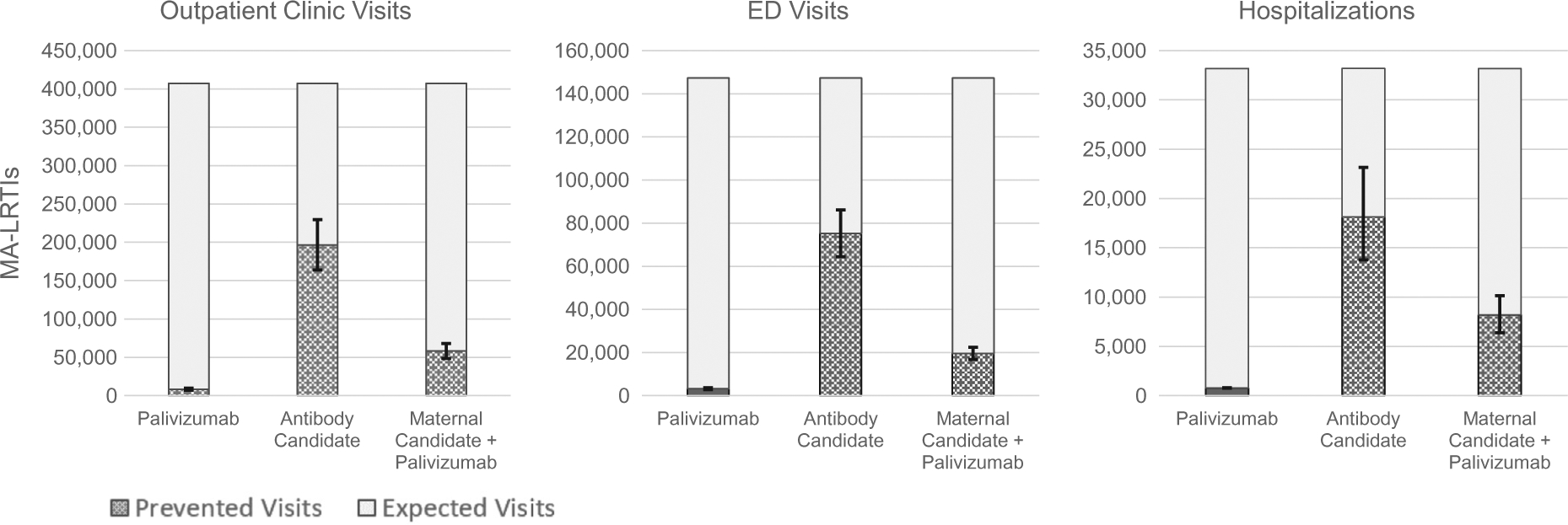
Estimated number of LRTI Visits Expected without Immunization and Prevented by Immunization in the US, by Healthcare Setting and Immunization Strategy. Error bars reflect uncertainty in the number of prevented MA-LRTIs associated with uncertainty in RSV rates. Uncertainty in the expected visits despite immunization is not shown.

In our illustrative scenario, Strategy II (the “Antibody Candidate”) prevented the most annual LRTIs. (Fig. 1) This strategy prevents an estimated 196,470 (48% of visits without immunization) RSV-associated LRTIs attended in the outpatient clinic setting (range: 163,810–229,650), 75,250 (51%) LRTIs attended in the ED (range: 64,430–86,090), and 18,140 (55%) LRTI hospitalizations (range: 13,770–23,160). Strategy III (the “Maternal Vaccine Candidate + Palivizumab”), prevented an estimated 58,210 (14% of visits without immunization) RSV-associated LRTIs attended in the outpatient clinic setting (range: 48,520–67,970), 19,580 (13%) LRTIs attended in the ED (range: 16,760–22,400), and 8190 (25%) LRTI hospitalizations (range: 6,390–10,150). We estimate that Strategy I, (“Current US Recommendations”), prevents 8,460 (2% of visits without immunization) RSV-associated LRTIs attended in the outpatient clinic setting (range: 7,050–9,880), 3,240 (2%) LRTIs attended in the ED (range: 2,770–3,710), and 780 (2%) LRTI hospitalizations (range: 760–800).

### 3.3. Deaths with and without immunization

We estimated 33 deaths (range: 25–43) would occur annually among hospitalized infants in the US from RSV-associated LRTIs in the absence of immunization, and following current recommendations for palivizumab use (Strategy I) prevents just one death. Eighteen in-hospital deaths (range: 14–23) would be prevented if immunization were implemented according to Strategy II, and eight in-hospital deaths (range: 6–10) prevented with Strategy III.

### 3.4. Sensitivity analyses

**Fig. 2.**
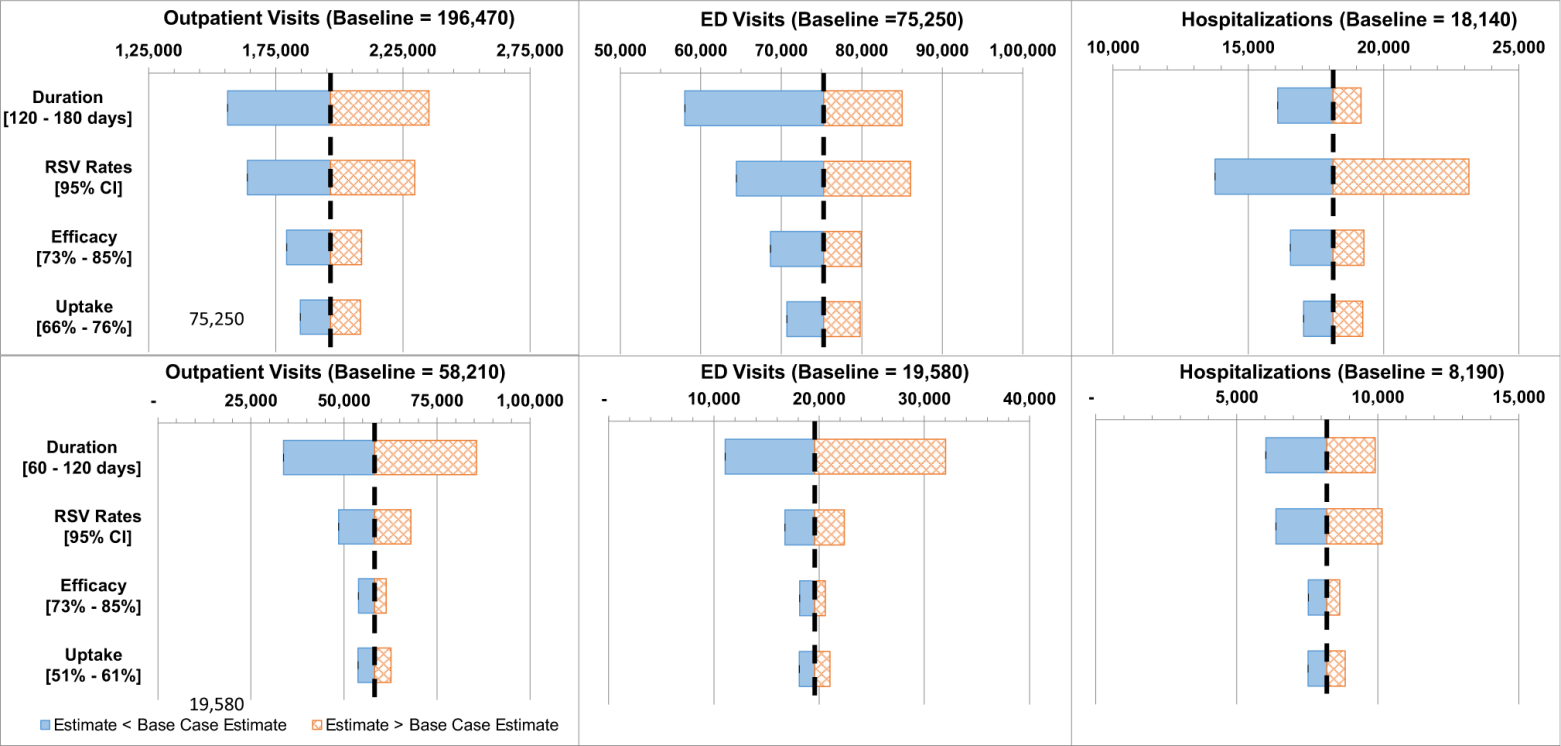
Sensitivity of Estimates of LRTI Visits Prevented to Select Model Parameters. Top row: Immunization Strategy II (the “Antibody Candidate” Strategy). Bottom row: Immunization Strategy III (the “Maternal Vaccine Candidate + Palivizumab” Strategy). Parameter values not shown, provided in Table 2.

The relative influence of individual parameters on our estimates of prevented LRTI-associated visits varied by immunization strategy and healthcare setting. In both Strategies II and III, uncertainty in the duration of immunization protection was the most influential parameter, except for the hospital setting, where uncertainty in RSV rates was more influential in Strategy II (Fig. 2). When results assuming 120 and 180 days of protection by the antibody candidate are compared, the estimated LRTI visits prevented differed by 3,080 in the hospital setting, 26,960 in the ED setting, and 79,070 in the outpatient clinic setting. When results assuming 60 and 120 days of protection by the maternal vaccine candidate are compared, the estimated LRTI visits prevented differed by 3,880 in the hospital setting, 20,950 in the ED setting, and 51,840 in the outpatient clinic setting. The more pronounced effects of immunization duration in Strategy III results from RSV rates peaking for the outpatient and ED setting at ages just after our baseline 90-day duration (Table S1). Antibody candidate uptake exhibited the least influence on prevented LRTIs in Strategy II. In contrast, efficacy was the least influential parameter in Strategy III.

The results of our multivariable sensitivity analysis suggest changes in the antibody candidate uptake have a larger impact in preventing RSV-associated LRTI visits than would uptake changes in the maternal vaccine candidate. For every percentage point increase in uptake of the antibody candidate, we estimate 1,435–3,527 outpatient visits would be prevented, compared with 273–1,611 for the same increase in the maternal vaccine candidate. In the ED setting, a one percentage point increase in antibody candidate uptake is associated with 548–1,248 LRTI visits prevented, while the same uptake increase in maternal vaccine candidate would prevent between 82 and 588 LRTIs. In the hospital setting, a one percentage point increase in antibody candidate uptake is associated with 128 to 329 prevented LRTIs, and 58 to 215 prevented LRTIs for the maternal vaccine candidate.

## 4. Discussion

Using the model and our best estimates of the parameters, we found that in the absence of an immunization, there are ~590,000 MA-RSV LRTIs among US infants and that new interventions that target all infants may prevent between ~86,000 to ~290,000 of those visits. These results indicate substantial RSV morbidity and associated healthcare utilization due to serious RSVi may be averted with new products under development. Few deaths (8–18), however, are averted, since few deaths in the US are attributed to RSVi. Of the candidates evaluated, administering an antibody candidate to all infants born during the season and at the season’s start for those born outside the season, prevents the most MA-LRTIs. With this strategy, we estimate nearly 200,000 outpatient clinic visits, 75,000 ED visits, and 18,000 hospitalizations for LRTIs could be prevented annually; approximately 48–55% (across settings) of visits estimated to occur without immunization. Our baseline estimates suggest this strategy may avert approximately 3.5 times the number visits for RSV-associated LRTIs to outpatient clinics and EDs, and two times the hospitalizations than a strategy in which a maternal vaccine candidate is offered to mothers year-round (in addition to palivizumab use per current US recommendations).

In our illustrative scenario, the difference in the number of prevented visits associated with candidates was largely attributable to the maternal candidate’s duration of protection being less than the antibody candidates’ duration of protection. This was especially pronounced in the outpatient clinic and ED settings, where the peak of incidence is beyond the 90 days of protection assumed for the maternal candidate. Consequently, changes to our duration assumptions for the maternal vaccine candidate had the greatest influence on product impact. Despite its lower impact, the maternal vaccine candidate has the potential to reduce MA-RSV LRTIs across all three settings by ~74,000 visits a year (beyond the ~12,500 visits prevented by palivizumab in our baseline scenario). Preliminary results suggest the efficacy of a maternal vaccine may be half what we assumed in our baseline estimates [20]. This would reduce visits prevented by the maternal vaccine candidate by about half, but not change the overall conclusion about the relative merits of the products and strategies evaluated.

Although uncertainty in factors over which public health practitioners have some influence, like uptake, had less impact on results, they were not trivial. For example, our multivariable sensitivity analysis suggests a 10% increase in uptake of the antibody candidate is associated with preventing an additional 14,350 to 35,270 outpatient clinic visits, 5,480–12,480 ED visits, and 1,280 to 3,290 hospitalizations for LRTIs. Similarly, a 10% increase in maternal vaccine candidate uptake is associated with preventing 2,730–16,110 outpatient clinic visits, 820–5,880 ED visits, and 580–2,150 hospitalizations for LRTIs. We also examined the influence of the timing of maternal vaccine uptake, by altering the immunization schedule so that it optimized the proportion of infants to whom antibodies successfully transfer (S2). The difference between these results and our baseline results were negligible.

The relative impact of strategies on hospitalizations are similar to Cromer et al.’s findings (ED and outpatients are not comparable) [13]. If we assume 100% uptake for both candidate products and limit our evaluation to infants <6 months of age (to match Cromer et al.’s analysis) we find the antibody candidate prevents 1.7 times more hospitalizations than the maternal vaccine candidate, compared with a ratio of 1.8 in Cromer et al. Our findings are also in line with previous studies examining the effect of a single type of vaccine with similar characteristics to products we examined. For example, Regnier, using a decision tree model to examine a theoretical vaccine for protecting infants in the US from birth, also estimated a 25% reduction in hospitalizations, but with assumptions of 69% uptake, 50% efficacy, and a decaying exponential distribution for the duration of protection with a 12 month median length [17]. And Hogan et al., employing a compartmental transmission model to examine maternal vaccine impacts in Western Australia, similarly estimated a 25% reduction in hospitalizations when assuming a similar immunization scenario of 50% uptake, 80% efficacy, and 3 months duration of protection [14]. A strength of our study is its simplicity. We focus on the impacts of products on infants who are actually immunized, which will be of specific interest to policy-makers developing RSV immunization guidelines. We do not estimate the indirect effects of immunization in infants (i.e. secondary infections prevented). However, this should not be seen as a limitation. Even Hogan et al. concluded from their transmission model that herd effects due to the maternal vaccine were modest and a simple cohort model would be a reliable alternative for estimating immunization impacts among infants [14]. Additional strengths of our study include evaluation of multiple candidate products, the separate consideration of infants with higher risk of healthcare use for RSV infection and the additional evaluation of the outpatient and emergency department settings.

RSV I^2^M has limitations. Estimates of immunization impact are restricted to the season in which they are given. It is possible that these products will shift the demand for care to subsequent seasons, although there is evidence that primary infection with RSV beyond 12 months of age is less likely to result in an LRTI [21]. We also do not account for the possible protection of mothers against RSVi by the maternal vaccine candidate. As such, and because we do not account for herd effects, we may underestimate the actual benefit of immunizing mothers. Other limitations, however, may result in our overestimation of immunization benefits. For example, our assumption that effective immunization averts healthcare use does not account for the potential that some portion of immunized infants may still become infected with RSV, but require a lower level of care (e.g. shift from hospitalization to outpatient visit). We also assumed an additive effect of palivizumab on top of visits prevented by the maternal vaccine candidate in Strategy III, on the basis that the population of “high-risk” births may derive partial protection from the maternal vaccine and from palivizumab. Any overestimation from this limitation, however, is negligible (in the US at least), since < 1% of births are affected. For jurisdictions that do not use palivizumab or who wish to see the potential impact of the maternal vaccine alone, users can set palivizumab uptake to 0%. It is worth noting that similar flexibility exists for analyzing impacts by setting: jurisdictions wishing to evaluate only the hospital setting can just input rates for this setting.

### 4.1. Conclusions

Our model provides decision makers with the ability to examine the impact of directly or indirectly immunizing infants against RSV infection with anticipated immunization products. As such, local and national public health agencies may use it to evaluate jurisdiction-specific scenarios of impact. The findings can be used in economic analyses to understand the direct costs and benefits of these strategies and others. The results of our illustrative scenario underscore potential for these products to reduce serious RSV illness and the benefits of each. Although we found limited impact of these products on deaths averted in the United States, they may have greater impact in places where RSV-associated deaths are more common. As more data become available regarding immunization candidates (i.e. study results regarding efficacy and length of protection) and the burden of RSV infections, our tool permits rapid updating of results.

## Supplementary Material

RSV Immunization Impact Model

Technical Appendix

Uncrestricted Open Access Copyright

## Figures and Tables

**Table 1:** Immunization Strategies.

	Strategy I	Strategy II	Strategy III	
Immunization Products	Palivizumab (licensed)	Antibody Candidate	Maternal Vaccine Candidate	Palivizumab (licensed)
Eligibility	High-risk infants*	All infants	All pregnant women^¶^	High-risk infants*
When offered	Within-RSV season	Within-RSV season	Year-round	Within-RSV season
Administration Schedule	Monthly injections for 5 months	Single injection	Single injection	Monthly injections for 5 months
Age when immunization initiated	Within-season birth: at birth Out-of-season birth: age at season’s start (1–6 months)	Within-season birth: at birth Out-of-season birth: age at season’s start (1–6 months)	3rd trimester of mother’s pregnancy	Within-season birth: at birth Out-of-season birth: age at season’s start (1–6 months)

* High risk conditions include hemodynamically significant congenital heart disease (CHD), chronic lung disease of prematurity (CLD), and prematurity (<29 weeks gestation) without CHD or CLD.

¶ Risk status of infant is not known at time of immunization.

**Table 2:** Inputs and Parameter Values for All Scenarios of Product Impacts in the US.

Parameter	Baseline Value	Range (used in sensitivity analyses)	User-adjustable	Source
**Population and Epidemiological**				
Annual live births	3,945,975		Yes	[22]
Births with conditions putting them at “high-RSV risk”^§^	0.98%		Yes	[23]
Percent of “high-risk” hospitalized before 12 months	9.31%		Yes	Calculated, S2
Rates of Medically-Attended RSV (per 1000 births)			Yes	See S2 for data
Hospitalizations	8.4	(1.5–30.8)		tables/sources
Emergency Department (ED) Visits	66.2	(16.8–132.7)		
Outpatient Clinic Visits	230.9	(71.0–337.2)		
Proportion of MA-RSVi visits with an LRTI diagnosis, by 0–5/5–11 months of age categories^^^			Yes	CDC/unpublished
Hospitalizations	1.00/1.00			
ED Visits	0.65/0.50			
Outpatient Clinic Visits	0.65/0.30			
Case fatality ratios^†^			Yes	[1]
0–5 months (%)	0.10			
5–11 months (%)	0.10			
RSV season	October-March		Yes	CDC/unpublished; S2
**Immunization**				
Uptake^#^				
Palivizumab	38.0%		Yes	[24,25]
Antibody Candidate				
Low-risk	71%	(66–76)	Yes	Assumed, [26,27]**
High-risk	80%		Yes	Assumed, [23]^¶¶^
Maternal Vaccine Candidate	56%	(51–61)	Yes	Assumed, [28]^‡‡^
Antibodies proportion successfully transferred to infants	91.9%		Yes	Calculated, S2
Efficacy (associated with full immunization dosage)				
Palivizumab	51%		Yes	[29]^§§^
Antibody Candidate	80%	(73–85)	Yes	Assumed, [30]^^^^
Maternal Vaccine Candidate	80%	(73–85)	Yes	Assumed, [30]^^^^
Duration of Protection				
Palivizumab	150 days		No	[31,32]
Antibody Candidate	150 days	(120–180)	Yes	[10]
Maternal Vaccine Candidate	90 days	(60–120)	Yes	[11]

¶ Illustrative average (unadjusted) population rates. S2 contains the actual age-based (monthly) rates used in all analyses.

§ High risk conditions include hemodynamically significant Congenital Heart Disease (CHD), Chronic Lung Disease of Prematurity (CLD), and Prematurity (<29 weeks gestation) without CHD or CLD.

^ Based on the average of number of lab-confirmed RSV visits from a national surveillance system between 2002 and 2009 with any of the following diagnoses: croup, bronchiolitis, bronchitis, pneumonia or asthma.

† Based on estimates for “high income/industrialized” countries.

# Percent of eligible population targeted to receive an immunization product that actually obtains and completes the full regimen. For Palivizumab: One injection monthly for 5 months on time. For Antibody & Maternal Vaccine Candidates: One injection.

** Baseline value is based on similar uptakes for Hepatitis B vaccine in neonates [27] (applicable to births within the RSV season) and Influenza immunization coverage among 6 month to 4 year olds [26] (applicable to births occurring outside RSV season). Range is −/+ 5 of baseline in the absence of data.

¶¶ Based on the percent of births that obtained the 1st palivizumab injection [23].

‡‡ Baseline based on average TdaP (tetanus, diphtheria, pertussis) uptake among pregnant women during a 15 month study period from April 2013 - June 2014 [28]; and range is −/+ 5 of baseline. TdaP, like the maternal RSV vaccine, is given in the 3rd trimester of pregnancy.

§§ This is the efficacy associated with our assumed uptake (i.e. compliance with all doses) [29].

^^ Based on average efficacy for term infants across all healthcare settings (hospitalizations, ED, and outpatient clinics) in a study examining the efficacy of motavizumab and our assumption of similarity between it and this study’s antibody and maternal vaccine candidates [30].

## Abbreviations:

RSVi: RSV infections
MA: Medically-attended
LRTI: Lower respiratory tract infection
