# Supplementary material for: Estimating the impact of multiple immunization products on medically-attended respiratory syncytial virus (RSV) infections in infants
Source: Vaccine. Author manuscript; Available in PMC 2020 Feb 19. (PMC7029767; doi:10.1016/j.vaccine.2019.10.023)
Supplement: Technical Appendix [file NIHMS1552870-supplement-Technical_Appendix.docx]

Supplementary File 2

## **This appendix provides further details on the methods used as well as additional results.**

**Illustrative Scenario Inputs**

Table 2 in the main text outlines the major inputs and parameter values used in this illustration. Our modeled annual birth cohort was 3,945,875, based on the 2016 Vital Statistics Report [22]. The proportion of high-risk births was based on a recent study that assessed palivizumab utilization [23]. Hospitalization rates for high-risk infants were based on hospitalization rates found amongst those who did not receive palivizumab in the original clinical trials [31, 32]. We obtained all-risk rates of MA-RSVi from population-based surveillance data published by the New Vaccine Surveillance Network (NVSN, Table S1) [4, 5]. We based the proportions of outpatient clinic and ED visits resulting in an LRTI on the average proportion of lab-confirmed RSV visits in NVSN from 2002-2009 with any of the following diagnoses: croup, bronchiolitis, bronchitis, pneumonia or asthma (CDC, unpublished). All hospitalized patients were assumed to have an LRTI. Case fatality ratios among hospitalized infants were based on estimates for high income/industrialized countries [1]. The RSV season was determined by the monthly distribution of outpatient, ED and hospital visits across NVSN during the 2000-2009 seasons (CDC, unpublished; Fig. S1). The estimate for palivizumab uptake (38%) is based on a recent study that defined compliance as receipt of all recommended doses [24, 25]. For the Antibody Candidate (anticipated to be a single injection), we assumed 71% uptake among low-risk newborns, based upon vaccination rates for the birth dose of hepatitis B vaccine [27], and 80% among high-risk newborns, which is an estimate of the percent of high-risk newborns that receive at least one dose of palivizumab [23]. For the Maternal Vaccine Candidate we based uptake (56%) on TdaP (tetanus, diphtheria, and pertussis) immunization in pregnant women because, like the maternal RSV vaccine, it is also given in the third trimester of pregnancy [28]. The efficacy used for palivizumab (51%) was based on a meta-analysis of randomized controlled trials among high risk infants where the endpoint was hospitalizations [29]. For the model, we assumed palivizumab reduces outpatient clinic and ED visits by the same percentage as hospitalizations. For the antibody candidate, we based efficacy (80%) against inpatient and outpatient MA-RSVi on findings from a clinical trial in term infants of an antibody product similar to palivizumab [30]. Since maternal vaccine also provides passive antibody protection to infants, we used the same efficacy for this product. We used 0.919 for the maternal vaccine reduction factor, which accounts for the percentage of infants acquiring antibodies successfully from their mothers (see S2 section “Maternal Vaccine Candidate”). Duration of protection was based on the endpoints of the clinical trials for each of the products (Table 2) [10, 11, 31, 32].

**Table S1**

US Population-based Rates per 1000 for Medically-Attended RSV infections by health care setting and month-of-age

|  | **Outpatient Clinic**  **Visits [5]** | | **Emergency Department**  **Visits [5]** | | **Hospitalizations [4]** | |
| --- | --- | --- | --- | --- | --- | --- |
| **Age (months)** | **Rate** | **95% CI** | **Rate** | **95% CI** | **Rate** | **95% CI** |
| 0 | 85.2 | (71.0-99.3) | 19.6 | (16.8-22.4) | 13.5 | (10.3-17.1) |
| 1 | 187.9 | (156.6-219.1) | 64.2 | (54.9-73.4) | 25.9 | (21.3-30.8) |
| 2 | 234.2 | (195.2-273.1) | 72.4 | (62.0-82.9) | 14.3 | (11.1-17.8) |
| 3 | 232.6 | (194.0-271.3) | 105.2 | (90.1-120.4) | 10.3 | (7.7-13.5) |
| 4 | 265.0 | (221.0-309.1) | 116.0 | (99.3-132.7) | 8.9 | (6.3-11.8) |
| 5 | 289.2 | (241.1-337.2) | 71.3 | (61.1-81.6) | 4.8 | (2.9-7.0) |
| 6 | 264.7 | (220.7-308.7) | 81.8 | (70.1-93.6) | 4.1 | (2.5-6.2) |
| 7 | 207.2 | (172.8-241.7) | 56.1 | (48.0-64.2) | 5.6 | (3.6-8.0) |
| 8 | 277.8 | (231.7-324.0) | 55.6 | (47.6-63.5) | 3.4 | (1.8-5.2) |
| 9 | 227.2 | (189.4-264.9) | 55.6 | (47.6-63.6) | 3.8 | (2.1-6.0) |
| 10 | 241.7 | (201.5-281.8) | 40.4 | (34.6-46.2) | 3.7 | (2.0-5.7) |
| 11 | 258.1 | (215.2-301.0) | 55.6 | (47.6-63.6) | 2.9 | (1.5-4.8) |


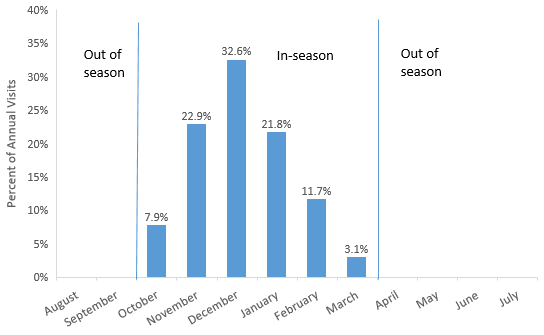


**Fig. S1.** Seasonal Distribution of Medically-Attended RSV infections in the US (National-level). Source: CDC, unpublished

**“High-Risk” births and RSV risk**

In our analysis “high-risk births” refers to infants who are recommended to receive palivizumab under current guidelines [8] and in our strategies I and III. In our illustrative scenario we calculated that 0.98% of all births are eligible to receive palivizumab in the US [23]. 0.98% is the sum of the percent of all births with the following three conditions associated with elevated RSVi risk: 1) hemodynamically significant congenital heart disease (CHD), 2) chronic lung disease of prematurity (CLD), and 3) prematurity (defined as <29 weeks gestation) without CHD or CLD (Table S2). We then obtained the hospitalization rates for each high-risk condition from hospitalization rates found amongst those who did not receive palivizumab in the original clinical trials [31, 32]. Next, we multiplied the percent of infants with each condition who are hospitalized with RSV by the proportion of all US births with the condition to determine the % of all live births for each high-risk condition that are hospitalized (Table S2). The sum of this product across all three conditions was 0.0913% and represents the percentage of live births with a high-risk condition that are hospitalized due to RSV. RSV I^2^M permits users to redefine high-risk conditions as they wish, accounting for up to four such conditions.

**Table S2**

Conditions Associated with Elevated Risk for RSV Infection and Percent Hospitalized

| **High-Risk condition** | **%, live births [23]** | **%, with condition hospitalized for RSV [31, 32]** | **%, live births with condition hospitalized for RSV*** |
| --- | --- | --- | --- |
| Hemodynamically significant congenital heart disease (CHD) | 0.39% | 9.7% | 0.0378% |
| Chronic lung disease of prematurity (CLD) | 0.12% | 12.8% | 0.0154% |
| Prematurity (<29 weeks gestation),  without CHD or CLD | 0.47% | 8.1% | 0.0381% |
| % of live births with a high-risk condition hospitalized due to RSV (column sum)🡪 | | | 0.0913% |

* Product of row data in prior two columns

**Proportion of high and low risk infants**

Although there was a placebo-controlled trial that showed palivizumab reduces MA-RSVi without hospitalization in preterm infants 33-35 weeks [33], there are no data available regarding the efficacy of palivizumab in reducing MA-RSVi in the outpatient clinic and ED settings among infants we define as “high-risk”. Therefore, we assumed the ratio of rates between high- and low-risk infants in the outpatient clinic and ED settings is the same as the ratio of hospitalization rates for high- and low-risk infants, and that palivizumab would have the same efficacy for preventing cases in these settings. We obtained the ratio of hospitalization rates for high- and low- risk infants by subtracting the expected number of high-risk hospitalizations from the expected number of all-risk hospitalizations. Expected all-risk hospitalizations were obtained by multiplying the birth cohort times the age-based rates in Table S1 and expected high-risk hospitalizations were obtained by multiplying the birth cohort times the percent of live births with a high-risk condition hospitalized due to RSV (0.0913%, from Table S2). These calculations resulted in a ratio of 8.4 low-risk visits for every high-risk visit (range 5.9 to 11.4, when using the 2.5 and 97.5 percentiles values from Table S1 for all-risk rates).

**Fig. S2.** Example Decision Tree Model Schematic. This schematic illustrates one of the decision trees used to track monthly birth cohorts in RSV I^2^M. This particular tree schematic is for within-RSV-season births evaluating Strategy II. Different tree structures were used for births occurring out of RSV season and the other evaluated Strategies. All trees are accessible in the RSV I^2^M modeling tool (S1). The probabilities associated with branching of a cohort are described in the main methods text (Table 2).

**Maternal Vaccine Candidate: Proportion of infants successfully immunized**

In our model, the percentage of antibodies that successfully transfer from mothers receiving the maternal vaccine candidate was dependent upon a combination of the timing of vaccination relative to the infant’s birth, and the infant’s gestational age at birth. We assumed there would be a partial (50%) transfer two weeks post-vaccination and full (100%) transfer by four weeks post-vaccination, and that maternal antibodies would not wane prior to birth (Table S3) [34, 35]. Additionally, we took into account the fact that the efficiency of placental transfer of antibodies is dependent on gestational age. We assumed that the amount of antibody transfer would be ineffective before 33 weeks gestational age and only partially effective (50%) between 33-36 weeks gestational age. At term, the transfer would be fully effective [36]. Taking into account the distribution of gestational ages of when births occur [22] and when mother’s receive TdaP vaccination (CDC, unpublished), we calculated that ~92% of antibodies that are needed to protect the cohort of infants would be successfully transferred across the immunized population. Since the protective level of antibodies needed to offer full protection has not been established we used this value as a proxy reduction factor for maternal vaccine efficacy. That is, we multiplied the percent protected by maternal vaccine by 0.92 to obtain the overall efficacy of maternal vaccine.

Table S3.

Proportion of maternal vaccinations with successful transfer of antibodies to the infant, by gestational age at immunization and birth

* calculated by 1) summing the product of the percent of births [22] and the proportion of antibody transfer in each row, then 2) multiplying the result by the percent of vaccinations administered for a given gestational age (percent value at top of each column, based on TdaP uptake [CDC, unpublished]), and 3) taking the sum of the column totals on the bottom row. This results in a total 91.9% successful transfer rate.

We also conducted a sensitivity analysis examining the influence of our assumption regarding the timing of immunization on the proportion of infants to whom antibodies successfully transfer. In this analysis we altered the immunization schedule so that it optimized the proportion of infants to whom antibodies successfully transfer. This was achieved when 100% of mothers receiving the maternal vaccine candidate are immunized when the fetus is exactly 29 weeks of gestational age. This improved the proportion of infants to whom antibodies successfully transfer by 2.1 percentage points to 94.0%. When this transfer proportion was used in our baseline illustrative scenario for the US (and all other parameters values in Table 2 remained the same), our estimated number of MA-LRTI hospitalizations prevented by the maternal vaccine candidate increased by 170 (from 8,190 to 8,360), prevented MA-LRTI ED visits increased by 380 (from 19,580 to 19,960), and prevented MA-LRTI outpatient clinic visits increased 1,140 (from 58,210 to 59,350).

References

1. Shi, T., et al., *Global, regional, and national disease burden estimates of acute lower respiratory infections due to respiratory syncytial virus in young children in 2015: a systematic review and modelling study.* Lancet, 2017. **390**(10098): p. 946-958.

2. Hall, C.B., et al., *The burden of respiratory syncytial virus infection in young children.* N Engl J Med, 2009. **360**(6): p. 588-98.

3. Byington, C.L., et al., *Respiratory syncytial virus-associated mortality in hospitalized infants and young children.* Pediatrics, 2015. **135**(1): p. e24-31.

4. Hall, C.B., et al., *Respiratory syncytial virus-associated hospitalizations among children less than 24 months of age.* Pediatrics, 2013. **132**(2): p. e341-8. doi: 10.1542/peds.2013-0303. Epub 2013 Jul 22.

5. Lively, J.Y., et al., *Respiratory Syncytial Virus-Associated Outpatient Visits Among Children Younger Than 24 Months.* J Pediatric Infect Dis Soc, 2019.

6. Rose, E.B., et al., *Respiratory Syncytial Virus Seasonality - United States, 2014-2017.* MMWR Morb Mortal Wkly Rep, 2018. **67**(2): p. 71-76.

7. Haynes, A.K., et al., *Respiratory syncytial virus circulation in seven countries with Global Disease Detection Regional Centers.* J Infect Dis, 2013. **208 Suppl 3**: p. S246-54.

8. AAP, *Red Book*. 31st ed. Report of the Committee on Infectious Diseases, ed. D.W. Kimberlin, et al. 2018, Itasca, IL: American Academy of Pediatrics.

9. PATH. *RSV Vaccine and mAb Snapshot*. 2018 [cited 2018 July 6]; Available from: https://vaccineresources.org/files/RSV-snapshot-2018Dec_High%20Resolution%20V3.pdf.

10. MedImmune. *A Study to Evaluate the Safety and Efficacy of MEDI8897 for the Prevention of Medically Attended RSV LRTI in Healthy Preterm Infants. (MEDI8897 Ph2b)*. 2016 [cited 2018 September 4]; Available from: https://clinicaltrials.gov/ct2/show/NCT02878330?term=MEDI8897&cond=RSV+Infection&rank=1.

11. Novavax. *A Study to Determine the Safety and Efficacy of the RSV F Vaccine to Protect Infants Via Maternal Immunization*. 2015 [cited 2018 September 4]; Available from: https://www.clinicaltrials.gov/ct2/show/NCT02624947.

12. Acedo, L., et al., *Mathematical modelling of respiratory syncytial virus (RSV): vaccination strategies and budget applications.* Epidemiol Infect, 2010. **138**(6): p. 853-60.

13. Cromer, D., et al., *Burden of paediatric respiratory syncytial virus disease and potential effect of different immunisation strategies: a modelling and cost-effectiveness analysis for England.* Lancet Public Health, 2017. **2**(8): p. e367-e374.

14. Hogan, A.B., et al., *Potential impact of a maternal vaccine for RSV: A mathematical modelling study.* Vaccine, 2017. **35**(45): p. 6172-6179.

15. Pan-Ngum, W., et al., *Predicting the relative impacts of maternal and neonatal respiratory syncytial virus (RSV) vaccine target product profiles: A consensus modelling approach.* Vaccine, 2017. **35**(2): p. 403-409.

16. Poletti, P., et al., *Evaluating vaccination strategies for reducing infant respiratory syncytial virus infection in low-income settings.* BMC Med, 2015. **13**: p. 49.

17. Regnier, S.A., *Respiratory syncytial virus immunization program for the United States: impact of performance determinants of a theoretical vaccine.* Vaccine, 2013. **31**(40): p. 4347-54.

18. Scheltema, N.M., et al., *Potential impact of maternal vaccination on life-threatening respiratory syncytial virus infection during infancy.* Vaccine, 2018. **36**(31): p. 4693-4700.

19. Yamin, D., et al., *Vaccination strategies against respiratory syncytial virus.* Proc Natl Acad Sci U S A, 2016. **113**(46): p. 13239-13244.

20. Novavax. *Phase 3 and beyond: The RSV F nanoparticle vaccine for infants via maternal immunization*. in *World Vaccine Congress*. 2019. Washington D.C.: https://novavax.com/download/files/posters/2019-World_Vaccine_Congress/2019.04.16-World_Vaccine_Congress.pdf.

21. Glezen, W.P., et al., *Risk of primary infection and reinfection with respiratory syncytial virus.* Am J Dis Child, 1986. **140**(6): p. 543-6.

22. Martin, J.A., et al., *Births : final data for 2016.* National vital statistics reports, 2018. **67**(1).

23. Pavilack, M., et al., *Trends in Utilization of Outpatient Respiratory Syncytial Virus Prophylaxis with Palivizumab among Medicaid- and Commercially Insured Infants.* Infect Dis Ther, 2017.

24. Stewart, D.L., et al., *Association of RSV-related hospitalization and non-compliance with palivizumab among commercially insured infants: a retrospective claims analysis.* BMC Infect Dis, 2013. **13**: p. 334.

25. Wong, S.K., et al., *Adherence and outcomes: a systematic review of palivizumab utilization.* Expert Rev Respir Med, 2018. **12**(1): p. 27-42.

26. *Estimates of Flu Vaccination Coverage among Children — United States, 2017–18 Flu Season*, in *FluVaxView*. 2018, Centers for Disease Control and Prevention (CDC).

27. Hill, H.A., et al., *Vaccination Coverage Among Children Aged 19-35 Months - United States, 2016.* MMWR Morb Mortal Wkly Rep, 2017. **66**(43): p. 1171-1177.

28. Healy, C.M., et al., *Tetanus and diphtheria toxoids and acellular pertussis vaccine uptake during pregnancy in a metropolitan tertiary care center.* Vaccine, 2015. **33**(38): p. 4983-7.

29. Andabaka, T., et al., *Monoclonal antibody for reducing the risk of respiratory syncytial virus infection in children.* Cochrane Database Syst Rev, 2013(4): p. Cd006602.

30. O'Brien, K.L., et al., *Efficacy of motavizumab for the prevention of respiratory syncytial virus disease in healthy Native American infants: a phase 3 randomised double-blind placebo-controlled trial.* Lancet Infect Dis, 2015. **15**(12): p. 1398-408.

31. Feltes, T.F., et al., *Palivizumab prophylaxis reduces hospitalization due to respiratory syncytial virus in young children with hemodynamically significant congenital heart disease.* J Pediatr, 2003. **143**(4): p. 532-40.

32. IMpact, *The IMpact-RSV Study Group. Palivizumab, a humanized respiratory syncytial virus monoclonal antibody, reduces hospitalization from respiratory syncytial virus infection in high-risk infants.* Pediatrics, 1998. **102**(3 Pt 1): p. 531-7.

33. Blanken, M.O., et al., *Respiratory syncytial virus and recurrent wheeze in healthy preterm infants.* N Engl J Med., 2013. **368**(19): p. 1791-9. doi: 10.1056/NEJMoa1211917.

34.       August, A. *RSVV F Vaccine: Phase 2 Clinical Trial to Protect Infants via Maternal Immunization*. in *FIGO World Congress of Gynecology and Obstetrics*. 2015. Vancouver.: <https://www.novavax.com/download/files/presentations/FIGO_7OCT2015_AA_P2_Data_10_14_15_FINAL_TO_USE.pdf>

35.       August, A., et al., *A Phase 2 randomized, observer-blind, placebo-controlled, dose-ranging trial of aluminum-adjuvanted respiratory syncytial virus F particle vaccine formulations in healthy women of childbearing age.* Vaccine. 2017 Jun 27;35(30):3749-3759. doi: 10.1016/j.vaccine.2017.05.045.

36. Palmeira, P., et al., *IgG placental transfer in healthy and pathological pregnancies.* Clin Dev Immunol, 2012. **2012**: p. 985646.

**Fig. S1.** Seasonal Distribution of Medically-Attended RSV infections in the US (National-level). Source: CDC, unpublished

**Fig. S2.** Example Decision Tree Model Schematic. This schematic illustrates one of the decision trees used to track monthly birth cohorts in RSV I^2^M. This particular tree schematic is for within-RSV-season births evaluating Strategy II. Different tree structures were used for births occurring out of RSV season and the other evaluated Strategies. All trees are accessible in the RSV I^2^M modeling tool (S1). The probabilities associated with branching of a cohort are described in the main methods text (Table 2)
