# Supplementary material for: Estimating the impact of multiple immunization products on medically-attended respiratory syncytial virus (RSV) infections in infants
Source: Vaccine. Author manuscript; Available in PMC 2020 Feb 19. (PMC7029767; doi:10.1016/j.vaccine.2019.10.023)
Supplement: Uncrestricted Open Access Copyright [file NIHMS1552870-supplement-Uncrestricted_Open_Access_Copyright.htm]

Rightslink® by Copyright Clearance Center


   


 
 

 


---

- Home
- Help
- Live Chat 

  Email Support
- Sign In
- Create Account

Estimating the
impact of multiple immunization products on medically-attended respiratory
syncytial virus (RSV) infections in infants

**Author:** 

Gabriel Rainisch,Bishwa Adhikari,Martin I. Meltzer,Gayle
Langley

**Publication:** 

Vaccine

**Publisher:**

Elsevier

**Date:**

10 January 2020

Copyright © 2020, Elsevier

Creative Commons

This is
an open access article distributed under the terms of the Creative Commons CC-BY license, which permits unrestricted
use, distribution, and reproduction in any medium, provided the original work is
properly cited.  
  
You are not required to obtain permission to reuse this
article.

To
request permission for a type of use not listed, please contact Elsevier
Global Rights Department.  
  
Are you the author of this Elsevier journal
article?

 
 

 

 
 


 
 


- © 2020 Copyright - All Rights
  Reserved
- Copyright
  Clearance Center, Inc.
- Privacy statement
- Terms and Conditions

  

Comments? We would like to
hear from you. E-mail us at customercare@copyright.com
